# The Effects of Growth Hormone Treatment Beyond Growth Promotion in Patients with Genetic Syndromes: A Systematic Review of the Literature

**DOI:** 10.3390/ijms251810169

**Published:** 2024-09-22

**Authors:** Anna Kucharska, Ewelina Witkowska-Sędek, Michał Erazmus, Dorota Artemniak-Wojtowicz, Maria Krajewska, Beata Pyrżak

**Affiliations:** Department of Pediatrics and Endocrinology, Medical University of Warsaw, 02-091 Warsaw, Poland; michal.erazmus@wum.edu.pl (M.E.); dorota.artemniak-wojtowicz@wum.edu.pl (D.A.-W.); maria.krajewska@wum.edu.pl (M.K.); beata.pyrzak@wum.edu.pl (B.P.)

**Keywords:** growth hormone treatment, genetic syndromes, metabolic effects, bone, muscle, QoL, children

## Abstract

Recombinant human growth hormone therapy (rhGH) has been widely accepted as the safe treatment for short stature in children with such genetic syndromes as Prader–Willi syndrome and Turner or Noonan syndrome. Some patients with short stature and rare genetic syndromes are treated with rhGH as growth hormone-deficient individuals or as children born small for their gestational age. After years of experience with this therapy in syndromic short stature, it has been proved that there are some aspects of long-term rhGH treatment beyond growth promotion, which can justify rhGH use in these individuals. This paper summarizes the data of a literature review of the effects of rhGH treatment beyond growth promotion in selected genetic syndromes. We chose three of the most common syndromes, Prader–Willi, Turner, and Noonan, in which rhGH treatment is indicated, and three rarer syndromes, Silver–Russel, Kabuki, and Duchenne muscular dystrophy, in which rhGH treatment is not widely indicated. Many studies have shown a significant impact of rhGH therapy on body composition, resting energy expenditure, insulin sensitivity, muscle tonus, motor function, and mental and behavioral development. Growth promotion is undoubtedly the primary benefit of rhGH therapy; nevertheless, especially with genetic syndromes, the additional effects should also be considered as important indications for this treatment.

## 1. Introduction

Recombinant human growth hormone (rhGH) therapy has been widely accepted as the safe treatment of short stature in genetic syndromes such as Prader–Willi syndrome (PWS) and Turner syndrome (TS). In many countries, government therapeutic programs for rhGH therapy in PWS and TS are available.

PWS results from the loss of paternally expressed imprinted genes on chromosome 15. In the majority of cases (65–75%), it is caused by the deletion of the paternally inherited chromosomal 15q11.2– q13 region; other cases are due to maternal uniparental disomy (20–30%) or due to imprinting defects and translocations of chromosome 15 [1]. The clinical picture is characterized by severe hypotonia and poor feeding in infancy, followed by hyperphagia with evolving obesity, decreased adult height, hypogonadism, and cognitive disabilities with behavioral disorders [1]. rhGH therapy was approved by the Food and Drug Administration (FDA) for use in PWS children in 2000 in the United States and 2001 in Europe. No consensus was reached on the age of the initiation of rhGH therapy in those children, although all authors agreed to the benefits of treating early in infancy, before the onset of obesity [2]. rhGH treatment is also recommended for PWS adults and should be continued after complete growth [3].

TS is one of the most common chromosomal aberrations, with an estimated incidence of approximately 25–50 cases per 100,000 women, caused by the complete or partial loss of one of the X chromosomes in all or some of the cell lines in females [4,5]. TS patients are mainly characterized by short stature, dysmorphic features, and gonadal dysgenesis. Untreated TS patients are approximately 20 cm shorter than healthy women [6,7]. Moreover, in this population, there is a more significant prevalence of congenital defects of the circulatory system, urinary tract, eye and hearing disorders, and autoimmune diseases. The haploinsufficiency of the SHOX gene is the main reason for short stature in TS girls, causing lower sensitivity to GH. Therefore, the doses of rhGH, according to most recommendations, are higher than in growth hormone (GH)-deficient individuals [5,6,7,8]. The optimal age to start treatment is 4–6 years of age [4,6,8,9,10].

The basic benefit of rhGH therapy is obviously to improve the final height of treated children; however, after years of experience with this therapy in syndromic short stature, it has been proved that there are some aspects of long-term rhGH treatment beyond growth promotion, which can justify rhGH use in these individuals. A further group of patients, such as children with Noonan syndrome (NS), Silver–Russel syndrome (SRS), Kabuki syndrome (KS), and Duchenne muscular dystrophy (DMD), may benefit from rhGH therapy. Nevertheless, in considering this therapy for such groups, it is important to analyze not only the potential benefits of long-term rhGH therapy but also possible risks and side effects.

NS is a genetic disorder caused by pathogenic variants of several protein-encoding genes involved in the RAS/mitogen-activated protein kinase (MAPK) signaling pathway [11,12]. Its prevalence is estimated to be between 1 in 1000 and 1 in 2500 live births [13]. The PTPN11, RIT1, SOS1, KRAS, RAF1, BRAF, and MEK1 gene mutations are found in approximately 70% of NS individuals [14,15]. The dysregulation of the RAS/MAPK signaling pathway leads to numerous changes in multiple extracellular signals, including hormones and growth factors, resulting in cardiac defects, energetic metabolism impairment, and increased risk for benign and malignant proliferative disorders [12,16,17]. NS children also present short stature (60–70% of NS children), facial dysmorphia, skeletal and ectodermal anomalies, and variable cognitive deficits [14,15,18,19]. GH abnormalities in NS children include growth hormone deficiency (GHD), GH insensitivity, and GH neurosecretory dysfunction [19]. rhGH treatment was approved by the FDA to accelerate growth and increase adult height for NS children in 2007 [20].

SRS is a rare, genetically, and clinically heterogeneous condition that occurs with a frequency between 1 in 30,000 and 1 in 100,000. It is associated mainly with 11p15 epimutation (20–60% of patients) and the maternal uniparental chromosome 7 disomy (7% to 15% of ones). However, the molecular etiology remains unknown in about 40% of cases with clinical features of SRS [21]. SRS children are characterized by intrauterine and postnatal growth retardation [22,23,24]. They exhibit a broad spectrum of dysmorphic and clinical symptoms such as body asymmetry, a triangular face with a small mandible, irregular and crowded teeth, a protruding forehead, and low muscle mass. Specific issues for SRS children include severe feeding difficulties, gastrointestinal problems, hypoglycemia, scoliosis, motor and speech delay, and psychosocial challenges [23,25,26,27,28,29,30,31].

KS is a genetic disorder caused by a mutation in the KMT2D (75% of cases) [32,33] or the KDM6A gene (9–13% of patients) [34]. Its prevalence is estimated at 1 in 32,000 (Japan). Clinically, it is characterized by postnatal growth retardation, facial dysmorphia, hypotonia, joint laxity, scoliosis, cardiovascular anomalies, hypertension, a variety of structural defects, and intellectual disability [35].

DMD is the most common hereditary X-linked neuromuscular disease, with a prevalence of 1 in 3500 male births. It is caused by maternal dystrophin gene mutations, which lead to muscle fiber degeneration and weakness in the skeletal, diaphragm, and cardiac muscle. Cardiac and orthopedic complications dominate the clinical picture. Most patients die due to cardiomyopathy or respiratory muscle weakness in the third or fourth decade of life. Current management options focus on glucocorticoid administration, considered the only therapeutic agent that can delay the progression of deterioration in muscle strength and motor function [36,37]. It has been confirmed that glucocorticoid therapy prolongs autonomous walking, improves respiratory function, reduces the incidence and progression of cardiomyopathy, and reduces scoliosis, which results in quality of life (QoL) improvement and life extension in DMD patients. On the other hand, they exert several side effects, such as growth retardation, osteoporosis, obesity, delayed puberty, and adrenal insufficiency [38].

It is well known that GH, except for growth promotion, exerts a number of metabolic effects acting directly or through the stimulation of insulin-like growth factor-I (IGF-I), insulin, and free fatty acids. It influences body composition, enhances muscle and bone mass accrual, promotes the achievement of an optimal peak bone mass, and affects carbohydrate, lipid, and protein metabolism. The GH effect on proteins includes increased protein synthesis and decreased breakdown in the whole body and in muscle, coexisting with a reduced degradation and oxidation of amino acids [39,40,41]. Clinical studies in children with GHD showed that long-term rhGH therapy exerts beneficial effects on body composition, could temporarily alter glucose metabolism in some individuals, and seems to have transient positive effects on lipid profile parameters [42,43,44,45,46,47]. The rhGH impact on carbohydrate metabolism mainly includes significant insulin level increases; some authors have also reported a substantial increase in fasting and oral glucose tolerance test (OGTT) serum glucose levels coexisting with high normal glycated hemoglobin (HbA1c) values [44,45,48,49].

Considering the effects of rhGH treatment, most reports focused on growth promotion, but the additional benefits could also be significant in specific groups of patients. This study aimed to show the current data about experience with rhGH treatment in selected syndromic short stature with a particular focus on the effects beyond growth promotion. The most well-known example of GH’s effects beyond growth promotion is its effect on body composition and bone density. Most analyses concern these two effects in different groups of patients treated with the growth hormone. Less well known is its effect on physical fitness and health-related quality of life.

## 2. Material and Methods

This study collected data from the literature about the syndromes in which the rhGH treatment is widely accepted: PWS and TS, and others for which patients could be treated if they meet the GHD criteria or are small for their gestational age (SGA), like NS, SRS, KS, and DMD. We did not include the Schaaf–Yang syndrome separately, taking into account its similarity to PWS.

The articles were searched in Pubmed/Medline and EMBASE. Systematic research was carried out, covering the years from January 2000 to December 2023, according to the Preferred Reporting Items for Systematic Reviews and Meta-Analysis (PRISMA) guidelines [50], using the Pubmed/Medline and EMBASE databases to identify studies reporting the effects of rhGH treatment beyond growth promotion in genetic syndromes in children and adolescents. The research found was based on the combination of the following keywords to generate a comprehensive search: (“Growth hormone treatment OR rhGH treatment OR rhGH therapy”) AND (“Genetic Syndrome OR Prader-Willi Syndrome OR Turner syndrome OR Silver-Russel syndrome OR Noonan syndrome OR RASopathies OR Kabuki syndrome OR Duchenne syndrome”) AND (“Pediatric OR Children OR Adolescent or infancy”).

The inclusion criteria were meticulously defined: articles written in English, belonging to the categories of clinical study, clinical trial, clinical trial protocol, multicenter study, randomized controlled trial, and observational study, which report the effects of long-term rhGH treatment in confirmed genetic syndromes in a pediatric population (age < 18 years). Due to the rarity of rhGH therapy in some genetic syndromes, like KS and DMD, case reports were also included, ensuring a comprehensive review of the available literature. However, it should be noted that case reports of single patients tended to describe the positive effects of treatment rather than its failures. The exclusion criteria were papers belonging to the review, systematic review, and meta-analysis categories; including only a non-pediatric population (age ≥ 18 years); and/or not evaluating the effects of rhGH therapy or confirmed genetic syndromes.

Initially, the research focused only on the genetic syndromes approved for rhGH therapy within the separate therapeutic program in Poland. Hence, TS and PWS were analyzed on a larger scale.

Two authors (A.K. and E.W.S.) screened the titles and abstracts of all retrieved articles to identify articles for full-text review. All authors assessed the eligibility of all full-text articles. Each author was responsible for analyzing their own thematic part.

## 3. The Results

**Figure 1:**
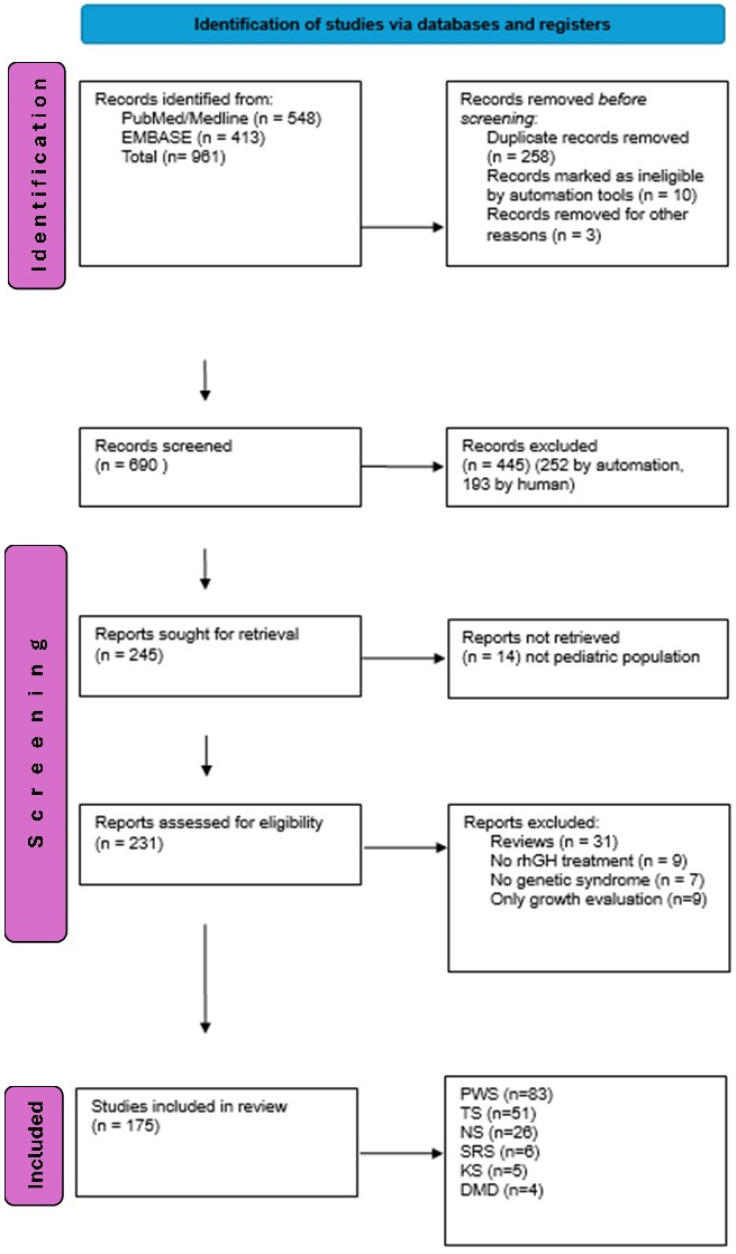
PRISMA 2020 flow diagram systematic reviews of rhGH therapy effects in children with selected genetic syndromes. PWS—Prader–Willi syndrome; TS—Turner syndrome; NS—Noonan syndrome; SRS—Silver–Russel syndrome; KS—Kabuki syndrome; DMD—Duchenne muscular dystrophy, rhGH—recombinant human growth hormone.

The initial search resulted in 961 references. Articles that did not report on GH treatment in genetic syndromes were excluded based on the title/abstract (445 articles). The remaining records were screened for inclusion criteria. After excluding duplicates (EndNote program and then manually), 231 potentially eligible studies were retrieved for full-text screening. For our final evaluation, we included 175 papers in the review (Figure 1). As expected, most of them concerned children with PWS, TS, and NS (83, 51, and 26 papers, respectively). A small number of papers focused on rhGH effects beyond growth promotion in SRS (*n* = 6), KS (*n* = 5), and DMD (*n* = 4) individuals.

The results were meticulously presented in accordance with the PRISMA guidelines [50], ensuring the highest standards of research reporting.

**Figure 2:**
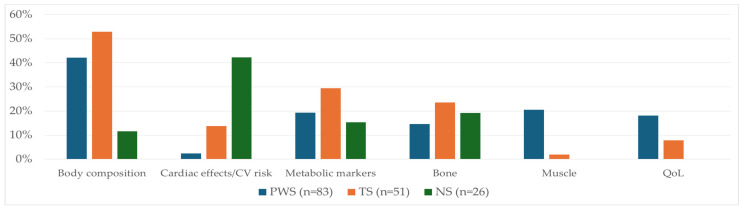
Comparison between percentage of papers regarding rhGH effects beyond growth promotion among PWS (% of 83 papers), TS (% of 51 papers), and NS (% of 26 papers). PWS—Prader–Willi syndrome; TS—Turner syndrome; NS—Noonan syndrome.

Finally, selected manuscripts (*n* = 175) were analyzed mainly in terms of the impact of rhGH therapy on body composition, cardiac function, cardiovascular (CV) risk, carbohydrate and lipid metabolism markers, bone, muscle, motor function, and QoL. The rhGH effects on body composition were the most frequently described in PWS children (35 of 83 papers, 42.2%) and TS girls (27 of 51 papers, 52.9%). The influence of rhGH therapy on the heart and/or on the CV risk was described mainly in NS children (11 of 26 papers, 42.3%) and TS girls (7 of 51 papers, 13.7%). Analyses of changes in carbohydrate and lipid metabolism markers and in rhGH effects on bone were reported in nearly similar percentages of papers on PWS, TS, and NS children (19.3%, 29.4%, and 15.4% and 14.5%, 23.5%, and 19.2%, respectively), whereas data concerning the effects of rhGH therapy on muscle and/or motor function and on QoL mainly came from PWS patients (20.5% and 18.1% of papers, respectively, in PWS vs. 2% and 7.8% in TS vs. no papers on these issues in NS individuals). The percentages of papers regarding the rhGH effects beyond growth promotion in PWS, TS, and NS are presented in Figure 2.

Description of the main rhGH effects on body composition, heart/CV risk, metabolic markers, bone structure, muscle/motor function, and QoL in each of the six analyzed genetic disorders are presented in Table 1.

## 4. Discussion: The Effects of rhGH Treatment beyond Growth Promotion in Genetic Syndromes

### 4.1. Prader–Willi Syndrome

Multiple studies with rhGH in children with PWS have reported improved final growth and a significant impact on body composition and resting energy expenditure. All studies reported a substantial reduction in fat mass (FM) with a simultaneous increase in lean body mass (LBM) during rhGH treatment [51,52,53,54,55]. This effect was correlated with the adiponectin level [56], its receptor expression [57], and leptin levels [58]. Some studies suggest that rhGH therapy in prepubertal PWS children could worsen insulin sensitivity, especially in obese individuals [59,60]. The mechanism of this effect contributes to leptin, adiponectin, neuropeptide Y, asprosin [61], and ghrelin [62,63,64,65]. In line with these observations were the results of the study of Bakker et al., who, in a randomized controlled trial, analyzed body composition and resting energy expenditure before and during rhGH treatment in prepubertal children with PWS [66,67]. Similar observations were reported by several authors in children [51,68] as well as in infants [53,69,70,71,72,73,74,75,76,77,78,79,80,81,82]. It is worth noting that in some studies, the body mass index (BMI) value did not change or was only slightly decreased without statistical significance. Nevertheless, the anabolic effect of rhGH on skeletal muscle, together with the lipolytic effect on subcutaneous and visceral adipose tissue, results in a better proportion of LBM and FM. Moreover, many studies have highlighted the advantages of the early initiation of rhGH therapy, before the nutritional phase of increased appetite, for improved body composition and nutritional status in PWS patients [2,83,84,85]. It could depend on many factors, such as decreased ghrelin secretion, which is the most important factor causing a central limitation of hyperphagia [54,86].

Increased LBM was associated with better muscle development and would be expected to increase exercise capacity. This was confirmed in several studies [87,88,89,90,91,92,93,94,95] concerning increased muscle volume and strength in PWS patients during the rhGH treatment and exercise tolerance improvement [91,96]. Moreover, Reus et al. [90] found a positive correlation between the initiation of rhGH therapy and the age at which infants start to walk.

The influence of rhGH was detected not only in skeletal muscle but also in the heart, where the rhGH treatment leads to left ventricular mass increase with the preservation of systolic and diastolic function [97]. Also, Hauffa et al. reported the effect of rhGH on cardiac dimensions in PWS children [98]. Considering the CV risk of rhGH in PWS, Manzardo et al., according to a questionnaire survey, reported thromboembolisms less often in PWS patients treated with rhGH than in individuals who had never been treated with rhGH [99]. The beneficial influence of rhGH on CV risk factors in PWS patients was reported in children after three years of treatment [100].

An essential aspect is the lipid profile alteration during rhGH treatment. A more protective lipid profile was reported in PWS patients who started rhGH therapy in infancy. Low-density lipoprotein (LDL) cholesterol was significantly lower, and the lipid profile and glucose metabolism parameters, including the homeostatic model assessment-insulin resistance (HOMA-IR), were better in treated patients. In the study of de Lind van Wijngaarden et al. [101], it was reported that there was a lack of influence of rhGH on LDL and triglyceride (TG) concentration; however, the high-density lipoprotein (HDL) cholesterol/LDL ratio was impaired before the therapy, and rhGH treatment improved the HDL cholesterol-to-LDL cholesterol ratio, but had no effect on serum lipids [101].

The next area of rhGH influence in PWS is bone tissue. Studies assessing the effect of rhGH treatment on bone suggest a positive effect on bone geometry, bone strength, and bone mass maintenance [102,103,104,105].

The local production of IGF-I in osteoblasts induced by rhGH is beneficial for bone tissue mineralization [106]. Bone formation and resorption markers, N-terminal propeptide of type I procollagen and osteocalcin, as well as other markers of bone remodeling, increased during rhGH therapy in PWS children [107,108]. Also, bone mineral density increased [109].

rhGH treatment in the context of bone and skeleton health in PWS was also analyzed, focusing on the risk of scoliosis development [110,111,112,113]. However, the authors agree that it should not be a contraindication to rhGH therapy, and long therapy could be even more beneficial, considering better motor function and a lower risk of scoliosis deterioration.

There is also some evidence for the influence of rhGH therapy on cognitive function and QoL in PWS patients. Cognitive function in PWS is related to the kind of genetic type [114]. Patients with uniparental disomy (UPD) usually have a higher verbal intelligence quotient (IQ) than patients with deletion (DEL). On the other hand, some studies are reporting lower psychological and academic achievements in DEL patients in comparison to UPD [115]. The studies that assessed the effect of rhGH treatment on cognition and QoL were inconclusive. Some of them found improvement in cognition and QoL [116], whereas others did not [117].

However, different research instruments were used across the studies, making comparing the results difficult. Nevertheless, there is some evidence that cognitive and motor development in children significantly improves when rhGH treatment is introduced early, i.e., between 3 and 9 months of life [84,95]. Moreover, rhGH therapy enhances final cognitive, linguistic, and motor development as well as adaptive function [109,118,119,120,121,122,123,124,125]. PWS children who started the therapy in infancy had higher nonverbal and total IQ scores and better adaptive communication skills in longitudinal observations [126].

The most controversial area is the respiratory function and the risk of deterioration of sleep-disordered breathing in PWS children after the start of rhGH therapy. Many studies were performed to explain this issue [127,128,129,130,131,132,133,134,135,136,137,138]. In the majority of them, it was suggested that the highest risk of obstructive sleep apnea happened in the initial period of the therapy and should be monitored thoroughly for the first two years after the initiation of rhGH treatment.

### 4.2. Turner Syndrome

There have been proven positive impacts of rhGH therapy on body composition, general development, socio-psychological aspects, and QoL, as well as on the circulatory system in TS patients [139]. Many studies have shown that treatment with rhGH reduces the amount of adipose tissue and increases LBM [9,140,141,142,143,144,145,146]. This is essential because patients with TS tend to have obesity. However, there are no data on whether this effect persists long term after the end of treatment. Some researchers suggest that this effect wears off relatively quickly in adult patients [9,141,143,147]. Several studies have also confirmed the beneficial effect of rhGH on the lipid profile, whereas the impact on glucose metabolism seems unclear [148,149,150,151,152,153]. Some authors suggest a slightly increased risk of insulin resistance [151,154,155,156,157,158]. It is additionally crucial among patients with TS who have an increased CV risk. Congenital heart defects such as bicuspid aortic valve, aortic regurgitation, and aortic coarctation are more common in this population, and their complications may worsen over time, especially in the presence of hypertension [159]. Data analysis highlights a decreased risk of ischemic disease during rhGH therapy [4,5,153,158,160]. However, some reports suggest that rhGH treatment could cause aorta widening or increase the aorta growth rate, and the level of IGF-I can be independently associated with increased aortic diameters [153,161]. Therefore, during rhGH therapy, monitoring the IGF-I concentration and systematically checking echocardiography is necessary [4].

Most studies and meta-analyses do not show a positive correlation between rhGH therapy and bone mass and bone mineral density (BMD) in TS girls [4,141,146,162,163,164,165,166,167,168]. However, the guidelines emphasize the beneficial effects on BMD, reducing the risk of osteoporosis in the case of combined rhGH therapy and estrogen supplementation, with the probable impact of synergy [169,170]. Some studies additionally highlight the beneficial effect of rhGH treatment on craniofacial development, with the most significant impact on posterior facial height and mandibular ramus [171,172].

In addition to the positive effect on final height and body composition, it has also been proven that treatment with rhGH in patients with TS has a beneficial effect on increasing muscle mass and muscle volume [5,141]. This effect could be enhanced by adding small doses of oxandrolone [4,5], which is used in some countries.

There is also evidence for the beneficial effect of rhGH treatment on the size of the uterus in pre- and peripubertal girls with TS before the implementation of estrogen therapy [173,174].

Many studies have proven that rhGH therapy has an extremely beneficial effect on the QoL of patients with TS; however, some analyses question the methodology of collecting data on this topic, as well as the final effectiveness of rhGH treatment without early estrogenization [5,139,175,176,177]. A better final height results in greater self-confidence, better integration, and better functioning in peer groups and later in adult life [147,148]. Also, rhGH therapy helps these patients function better in school groups and promotes better education, which improves their intellectual abilities and different skills in adulthood [4,5,175,178,179].

In analyzing the potential risks of therapy, it is worth mentioning single-case reports of the occurrence of malignancies in girls with TS treated with rhGH [180,181,182,183]. Still, the possible initial higher risk of neoplasms in TS itself is unclear. Another potential negative impact of rhGH therapy reported in a few studies was a risk of elevated liver enzymes in TS girls. However, this effect remains unclear and has no negative repercussions on liver function [184]. It is also essential to take into account that elevated liver enzymes in young TS patients were observed before sex hormone replacement therapy, and some studies showed a beneficial impact of estrogen replacement.

### 4.3. Noonan Syndrome

The growth-promoting effects and positive changes in bone metabolism and body composition of rhGH therapy in NS children, especially those with GHD, have been well documented. On the other hand, there are concerns that such a therapy could carry the risk of worsening cardiac function, potentially accelerating tumor risk in tissues expressing IGF-I receptors and impairing carbohydrates metabolism [11,16,185,186,187].

Clinical studies confirm positive effects of rhGH treatment on body composition in NS children [188,189,190] in whom a significant increase in fat-free mass and total body water accompanied by a decrease in percentage fat mass during rhGH therapy have been reported [188]. The study by Lee et al. [189], which analyzed the data of more than one hundred NS children included in the American Norditropin Studies, showed proportional increases in height and body weight during long-term rhGH administration, whereas BMI remained stable. Similar findings were presented by Zavras et al. [190], who confirmed that BMI values do not significantly change during the first five years of rhGH therapy. Delagrange et al. [191] revealed that muscle mass is significantly lower in NS children compared to healthy age-matched controls, even after adjusting for height; deteriorates with age; and correlates with low bone mass. Reduced muscle strength and endurance that lead to impaired motor performance were also reported in the NS group [192,193]. Even though data concerning effects of rhGH on muscle function in NS children are scant, it can be assumed that NS children may benefit from these kinds of treatment, especially in combination with increased physical activity during rhGH administration.

Cardiac abnormalities are observed in 70–80% of NS individuals. The most common are pulmonary valve stenosis and hypertrophic cardiomyopathy (HCM). Moreover, 50% of NS patients have electrocardiographic abnormalities, even those without structural heart defects [13,194]. Although most of the studies available do not show clinically significant adverse effects of rhGH therapy on left ventricular dimensions and cardiac function in NS children [185,186,190,195,196,197,198], some cases of HCM development, the worsening of existing HCM, and pulmonary valve-related events have been reported during rhGH therapy [187,199,200]. Ranke et al. [187], based on NS patients’ data collected for 25 years in the Pfizer International Growth Database (KIGS), reported that serious cardiac system adverse events, such as atrial fibrillation, left ventricular hypertrophy, and pulmonary valve stenosis were observed in less than ten individuals. All authors emphasize the need for careful cardiac monitoring during rhGH therapy.

The risk of glucose metabolism impairment during rhGH therapy in NS children does not seem to be higher than in the other rhGH-treated patients. Available studies show that in most NS children, both serum glucose levels and HbA1c values did not change significantly after the initiation of rhGH therapy [185,195,201]. The studies by Ogawa et al. [202] and Osio et al. [203] reported slight increases in glucose and insulin serum concentrations in rhGH-treated NS individuals. Data concerning the effects of rhGH therapy on lipid metabolism in NS children are limited. Some NS individuals, especially those with PTPN11 pathological variants, could present unfavorable lipid profiles with low HDL cholesterol and elevated TG levels irrespective of rhGH therapy [17,204]. Data presented by MacFarlane et al. [195] indicate that long-term rhGH therapy does not significantly affect cholesterol and TG levels in NS children.

Reduced BMD and lower axial and appendicular bone mass have been reported in NS children [191,205]. Delagrange et al. [191] suggested that bone loss observed in these individuals could be explained by a decrease in muscle mass and IGF-I deficiency. The study by Noordam et al. [188] showed that pre-treatment BMD values slightly increase over two years of treatment. Clinical studies also reported significant delay in bone age in NS children, usually accompanied by puberty delay, with acceleration during rhGH therapy [186,189,202,206]. Skeletal abnormalities in NS children include scoliosis, anterior chest wall anomalies, and hand anomalies [12]. The risk for scoliosis development or progression in NS children after the initiation of rhGH therapy seems to be non-significant, but data in this field are scant [12,16].

Some NS children could present developmental delay with clumsiness and poor coordination. IQ scores in NS individuals are lower than unaffected family members [207]. Arnold–Chiari malformation has also been reported in NS children, although the incidence is unknown [12]. Data concerning the influence of rhGH therapy on cognitive function, QoL, and the risk of worsening of Arnold–Chiari malformation are insufficient.

In NS individuals with mutated PTPN11, cancer risk is estimated to be 3.5 times higher than in the general population [208]. NS is associated with a higher risk for juvenile myelomonocytic leukemia, other hematological malignancies, and solid tumors, such as neuroblastoma, brain tumors, and embryonal rhabdomyosarcoma [12]. Data concerning the safety of rhGH therapy in NS children show no evidence to support a higher prevalence of neoplasm during such treatment [16], but careful clinical monitoring is recommended after rhGH administration. In the literature, there are only a few studies available reporting cases of intracranial or spine neoplasms in rhGH-treated NS children, but their associations with such therapy is considered unlikely and is difficult to confirm [16,19,187]. Jorge et al. [19] reported two cases of brain neoplasms in rhGH-treated NS children, the posterior fossa tumor with spine metastases and dysembryoplastic neuroepithelial tumor. Both patients were PTPN11-positive [19]. In the KIGS cohort, two individuals developed tumors [187]. Wu et al. [209] reported one case of osteochondroma in both the right and left tibia diagnosed six months after the initiation of rhGH treatment. Şıklar et al. [206] reported one case of non-ossifying fibroma that occurred while receiving rhGH treatment. Moos et al. [210] reported one case of an atypical granular cell tumor occurring in an individual with Noonan syndrome after three years of rhGH therapy.

### 4.4. Silver–Russel Syndrome

Numerous studies have shown that children with SRS who are born SGA and remain short benefit significantly from rhGH therapy [211,212,213,214,215,216]. They have been treated under the treatment program for children born SGA in the United States since 2001, and in Europe since 2003 [217]. Several studies have revealed that height improves with rhGH treatment in SRS children, although the final adult height attained in patients with SRS is lower than in those with non-syndromic SGA [215].

In addition to the growth effect, rhGH treatment in this group of children offers other benefits, like weight gain and increases in BMI [213,216,218]. An increase in BMI in SRS children, unlike for individuals with other syndromes, is considered an advantage because SRS patients are usually weight-deficient. It was observed that in SRS patients, during rhGH treatment, individual body composition parameters (LBM, FM, skeletal muscle mass, total body water) expressed in kilograms increased significantly, relatively to the increase in total body weight. Simultaneously the percentage of FM (%FM) decreased significantly, leading to body composition optimization [24,218]. This result is probably related to the catabolic effect of GH on adipose tissue [219]. However, Smeets et al. found the opposite results, in which %FM increased during rhGH therapy [220]. This topic requires further research.

In children with SRS under 5 years of age, the risk of fasting hypoglycemia is approximately 27% [24]. Some studies have shown that during rhGH therapy, fasting glucose levels increase, diminishing the risk of hypoglycemia [220,221].

Basing on the data obtained in patients treated with rhGH—GH-deficient children as well as patients with PWS or TS—one can expect that rhGH therapy in children with SRS can also lead to increased muscle mass, better bone mineralization, and improved QoL and perception skills; however, on SRS children, no study is available to support these observations. Despite the possible side effects, the advantages of rhGH therapy outweigh the harmful effects; thus, the international consensus recommends the early initiation of rhGH treatment for SRS [24].

### 4.5. Kabuki Syndrome

KS children are at risk of developing obesity, which can lead to metabolic syndrome in adulthood. Research on rhGH therapy effects in KS are scant. Shott et al. investigated the influence of one-year rhGH therapy in a group of 18 KS children and noticed that total energy expenditure significantly increased during the first six weeks of treatment [222]. Moreover, the increase was higher among children with lower levels of GH secreted during stimulation tests. The basal metabolic rate, fat-free mass, and physical activity level also increased significantly in the period of rhGH therapy. The results confirmed that growth hormone has an anabolic effect by increasing nitrogen retention, protein synthesis, and fat-free mass, which are associated with higher energy expenditure and positively affect body composition [222]. After one year of rhGH therapy, all KS patients had reduced their BMI, which remained stable as they continued the treatment in the following years [223]. It is worth noting that one year after the end of the two-year treatment, BMI significantly increased [223]. In children with obesity the weight standard deviation score increased in 14 children and decreased in 4 children [224]. There were also improvements in waist circumference and waist-to-height ratio (WHtR) at both one and two years of treatment [223,224]. In children with obesity, the WHtR decreased, but the result was not statistically significant [223]. No differences in KS children with or without obesity in their basal metabolic rate, total energy expenditure, and physical activity level before and during rhGH treatment were seen [223]. The results showed that rhGH therapy significantly improves body composition, regardless of baseline body weight, which promotes the prevention of obesity, which is often seen in KS children.

In the abovementioned studies, metabolic risk factors and cardiovascular markers were also investigated [223,224]. Researchers assessed the glucose and lipid profile, markers for endothelial function and low-grade inflammation-tumor necrosis factor-alpha (TNF-α), TNF receptors, interleukin (IL) 6, IL-8, and high-sensitivity C-reactive protein concentrations. Although none of the children had cardiometabolic abnormalities, metabolic syndrome, or hypertension at baseline, after twelve months of rhGH treatment, the researchers noticed a significant reduction in serum LDL cholesterol and pro-inflammatory cytokine IL-8 levels [224]. After two years of treatment, this relationship persisted in the LDL range, but not in IL-8 [223]. Moreover, authors reported a significant reduction in apolipoprotein (Apo) B-100 levels (major LDL apolipoprotein) and a decrease in the Apo-B100/Apo-A1 (the main component of HDL cholesterol) ratio, which may reduce the risk of myocardial infarction [223,224,225,226].

Blood pressure, fasting glucose, insulin, HbA1c, and HOMA-IR remained without significant changes after both one and two years of treatment [223,224]. There was a tendency toward insulin resistance after one year of treatment: both fasting glucose and insulin levels increased slightly. However, after two years of treatment, this trend no longer occurred [223].

Similarly, the markers of low-grade inflammation, despite the abovementioned IL-8 levels, did not change during rhGH treatment.

The results indicate that growth hormone therapy in children with KS improves lipid metabolism parameters.

Joint hypermobility, low muscle mass, and hypotonia negatively affect motor development in KS. The prevalence of joint laxity is described up to 75% in this syndrome. Shott et al. assessed the influence of two-year rhGH therapy on joint laxity in KS children and noticed a significant decrease in joint hypermobility and improvement in motor skills [227]. A potential way in which GH can cause joint stiffness is by triggering the production of decorin, a structural protein present in the extracellular matrix of the skeletal muscle, responsible for regulating the genes that control muscle growth and repair. Through this process, rhGH stimulates the synthesis of collagen in muscles and tendons [228].

### 4.6. Duchenne Muscular Dystrophy

Data concerning rhGH therapy in DMD patients are very limited, but some authors have postulated that rhGH therapy could be effective [221,229,230,231], primarily as a counterbalance to the side effects of therapy of glucocorticoids. It is well known that glucocorticoid administration impairs the GH/IGF-I axis leading to a reduction in GH secretion and increase in peripheral resistance to both GH and IGF-I [232]. On the other hand, the experimental study by Fang et al. indicates that glucocorticoids administered together with IGF-I promote myogenic differentiation through the Akt/GSK-3βpathway. The authors suggest that these results may offer a potential alternative strategy for DMD therapy based on glucocorticoid and IGF-I [233].

Cittadini et al. [229], in their pilot, double-blinded, placebo-controlled study, including six DMD patients with documented cardiac involvement, aimed to evaluate the effects of a three-month rhGH therapy on cardiac structure and function. The results showed a marked myocardial growth-stimulating effect in the rhGH treatment group, with a 29% increase in left ventricular mass, a 33% decrease in systolic stress, and a tendency to increase fractional shortening. This myocardial growth resulted in a significant reduction in circumferential wall stress, a tendency to improve systolic function, and no changes in diastolic filling or skeletal muscle function. A slight reduction in systemic vascular resistance was also found. These structural and hemodynamic changes led to a significant decrease in plasma b-type natriuretic peptide concentrations among the rhGH-treated group, which may reflect an improved hemodynamic profile. Throughout the treatment period, all DMD patients remained in the same Lown class, which is a grading system for ventricular premature beats, from 0 to 1. The cardiomyopathic index, which was abnormal in three DMD boys, did not change significantly. Cittadini et al. [229] also found that elevated IL-6 and TNF-α baseline levels remained unchanged at three months of rhGH treatment and postulated that high levels of those cytokines may contribute to the observed loss of impairment of the GH/IGF-I axis. The authors reported also that there were no differences in timed functional tests and no changes in pulmonary function tests or in the thyroid hormone concentrations [229].

The clinical study by Rutter et al. [230], evaluating the efficacy and safety of rhGH therapy in DMD boys with glucocorticoid-induced growth failure, showed that one-year rhGH administration led to a significant increase in height velocity with an unchanged rate of weight gain, cardiopulmonary function, and a similar decline in motor function. The mean BMI decreased with height gain, and lean body mass increased significantly during the first year of rhGH therapy, but the whole-body and truncal fat mass did not change significantly. The analysis of the metabolic profile showed that fasting glucose levels and HbA1c values remained normal after the initiation of rhGH therapy, but fasting insulin concentrations increased significantly. The authors emphasized that their study was not conducted to evaluate the effect of rhGH treatment on motor function and long-term prospective studies are needed to answer if there are any beneficial effects of such therapy in DMD [230].

Lavi et al. [231], who evaluated the outcome of rhGH therapy in four glucocorticoid-treated DMD boys reported that 6 to 18 months of rhGH therapy significantly improves height velocity without adverse events or deterioration in cardiac or respiratory parameters. They reported that the decline in motor function parameters did not change during rhGH therapy. Fasting glucose concentrations and HbA1c values remained within reference limits [231].

## 5. Conclusions

Research and observations regarding the metabolic effects of rhGH treatment collected from groups of patients with syndromic short stature treated in therapeutic programs may be the basis for expanding the indications for the use of rhGH for reasons other than growth promotion. Patients with some ultra-rare diseases often see rhGH therapy as a hope for improving their general condition, including physical and metabolic condition and life drive.

In our review, we presented the effects of rhGH treatment in genetic syndromes, for which this therapy is commonly recommended, and in those rare syndromes for which research remains sparse.

Growth promotion is certainly the basic benefit of rhGH therapy; nevertheless, in particular genetic syndromes, the additional effects of rhGH therapy should also be considered as important indications for this treatment to improve metabolic parameters, motor function, and QoL. PWS children rated health-related QoL equally or even better than healthy and obese children, and this rating was increased and sustained during long-term rhGH therapy. PWS children considered themselves quite happy, despite difficulties related to the syndrome. Similar observations were reported in TS girls treated with rhGH.

Further prospective studies assessing the benefits and risks associated with the use of growth hormone in patients with ultra-rare diseases, like DMD or KS, should be conducted to provide sound argumentation in this discussion.

## Figures and Tables

**Table 1:** The summary of the main rhGH effects beyond growth promotion in each of the six analyzed genetic disorders.

Name of Syndrome	Body Composition	Cardiac Effects/ CV Risk	Metabolic Markers	Bone Structure	Muscle/ Motor Function	QoL
PWS	↓ FM ↑ LBM no effect on BMI	↑ LV mass preservation of systolic/diastolic function ↓ CV risk ↓thromboembolism risk	↓ LDL ↑ HDL/LDL ↓ HOMA-IR	↑ bone geometry, strength, and mass ↑ BMD ↓ scoliosis risk	↑ motor function	↑ QoL ↑ cognition ↑ motor development ↑ nonverbal and total IQ
TS	↓ FM ↑ LBM	aorta widening or increase of the aorta growth rate *	improvement in lipid profile	↑ BMD	↑ muscle mass ↑ muscle volume	↑ QoL
NS	↓ FM no effect on BMI	no significant adverse effect on LV dimensions and cardiac function **	no effects or ↑ glucose/insulin no effect on lipid profile *	↑ BMD no effect on scoliosis risk	no data	no data
SRS	↓ or ↑ %FM ↑LBM	no data	↑ fasting glucose	no data	↑ muscle mass ↑ BMD	↑ QoL ↑ perception skills
KS	↑ LBM no effect or ↓ BMI	no data	↓ LDL ↓ IL-8 no effect on glucose/insulin	no data	↓ hypermobility of joints ↑ motor skills	no data
DMD	↑ LBM ↓ BMI	↑ LV mass ↓ systolic stress *	↑ fasting insulin no effect on glucose	no data	no effect on skeletal muscle function	no data

* limited data; ** reported cases of worsening of existing hypertrophic cardiomyopathy and pulmonary valve-related events. PWS—Prader–Willi syndrome; TS—Turner syndrome; NS—Noonan syndrome; SRS—Silver–Russel syndrome; KS—Kabuki syndrome; DMD—Duchenne muscular dystrophy, rhGH—recombinant human growth hormone; CV—cardiovascular; QoL—quality of life; FM—fat mass; %FM—% fat mass; LBM—lean body mass; BMI—body mass index; LV—left ventricle; LDL—low-density lipoprotein; HDL—high-density lipoprotein; HOMA-IR—homeostatic model assessment-insulin resistance; BMD—bone mineral density; IL—interleukin; IQ—intelligence quotient.

## Data Availability

Data are contained within the article.
